# The influence of lithium on hippocampal volume in elderly bipolar patients: a study using voxel-based morphometry

**DOI:** 10.1038/tp.2016.97

**Published:** 2016-06-28

**Authors:** S Zung, F L Souza-Duran, M G Soeiro-de-Souza, R Uchida, C M Bottino, G F Busatto, H Vallada

**Affiliations:** 1 Department of Psychiatry, University of Sao Paulo Medical School (LIM-21 and LIM-23), Sao Paulo, Brazil

## Abstract

Recent studies have demonstrated that lithium (Li) exerts neuronal protective and regenerative effects both *in vitro* and *in vivo*. However, the effects of long-term Li treatment in the brain areas associated with memory impairment of elderly bipolar patients are still unknown. The aim of this study was to compare the hippocampal volumes of elderly bipolar patients using Li, elderly bipolar patients not using Li and healthy controls. Sociodemographic, clinical and magnetic resonance imaging data from 30 elderly euthymic bipolar patients who had been using Li for an average of >61 months; 27 elderly euthymic bipolar patients not taking Li for an average of 45 months; and 22 elderly healthy controls were analyzed. Volumetric differences in the hippocampus between groups were investigated with voxel-based morphometry (VBM) based on the Statistical Parametric Mapping technique. No statistical differences in sociodemographic and clinical characteristics and course of bipolar disorder between the two bipolar groups were observed. Using small volume correction in the VBM analysis (analysis of variance (ANOVA)), one voxel cluster of statistical significance was detected in the left hippocampus (*P*<0.05 corrected for multiple comparisons, extent threshold >10 voxels). *Post hoc* unpaired *t*-tests revealed increased left hippocampal volume in the Li-treated group compared with the non-Li-treated group, and decreased left hippocampal volume in the non-Li group relative to controls. Additional exploratory two-group comparisons indicated trends toward reduced right-hippocampal volumes in the non-Li-treated group relative to both the Li-treated group and controls. The findings suggested that the use of Li may influence the volume of the hippocampus, possibly due to its neuroprotective effects.

## Introduction

Lithium (Li) is an alkali metal that began being used in medicine as a treatment for gout in 1840.^1^ It was later used widely in elixirs and tonics, as well as a salt substitute for cardiac patients. Due to severe toxicity, Li remained neglected until the observations of John Cade in the 1940s about its calming effect on guinea pigs and on psychotic patients.^2^ Over the following decades many reports confirmed Li salt as being the most effective drug for the treatment of bipolar disorder (BD).^3, 4^

BD is a major public health problem characterized by functional impairment and clinical comorbidities.^5^ Prospective longitudinal studies in BD patients demonstrate progressive dysfunction during the disease course and a number of clinical variables have been associated with disease progression.^6, 7, 8, 9^ These variables include the number of affective episodes and the presence of psychotic symptoms, both of which have been correlated with cognitive impairment, lower neurotrophic factors and brain atrophy.^6, 7, 8, 9, 10^

Chronic Li treatment may stimulate neuroprotection through several mechanisms: (1) attenuation of *N*-methyl-d-aspartate receptor-mediated excitotoxicity,^11^ (2) activation of factors promoting cell viability,^12^ (3) induction of the expression of cytoprotective proteins such as brain-derived neurotrophic factor^13^ and improvements in neurogenesis in animal and *in vitro* experiments.^14, 15^ Some investigators have postulated that these mechanisms could lead to Li having a therapeutic effect on cognitive function, especially on memory.^16, 17^ Moreover, magnetic resonance imaging (MRI) studies suggest that Li treatment in BD is associated with regional increases in gray matter (GM) volume in the hippocampus and amygdala^18, 19, 20, 21, 22, 23^ although some studies suggest a global effect as well.^24^ Although, the influence of Li on brain morphology is clear, the majority of the studies on this matter were performed on middle age subjects and there is a lack of information about the effect of long-term Li treatment in the elderly.

One brain region known to play a central role in memory function is the medial temporal lobe. Several investigations have shown that the hippocampal area in the medial temporal lobe is particularly important for the formation of new memories about experienced events (episodic autobiographical memory).^25^ In addition, this brain region has also received significant attention in mood disorder research. Recent studies have reported decreased volume of the hippocampus in patients with depression^26^ and in older patients with BD compared with controls.^27^

In the present study, we compared the volume of the hippocampus between three groups: elderly bipolar patients using Li, elderly bipolar patients not using Li and elderly healthy controls. We hypothesized that the use of Li would exert a significant influence on the volumes of the hippocampus of elderly patients using Li for at least 1 year compared with elderly patients not using this drug.

## Materials and methods

### Subjects

The sample was recruited according to three predefined sets of individuals. The first group comprised bipolar patients using Li as a mood stabilizer for at least 1 year; the second group comprised bipolar patients who had not used Li in the previous 8 months; and the third group comprised individuals presenting no history of psychiatric disorder and who had never used Li carbonate. All individuals were ⩾60 years of age (mean 68.7 years).

The bipolar patients were selected from two university psychiatric institutions from the city of Sao Paulo, Brazil (the Institute of Psychiatry of the University of Sao Paulo Medical School and the Mental Health Hospital of the Faculty of Medical Sciences of Sao Paulo), through medical records using BD criteria according to the International Classification of Diseases (ICD-10).^28^ The patients who were not euthymic, or who also had a diagnosis of other major psychiatric disorder or neurological diseases, or who had received electroconvulsive therapy treatment in the previous 6 months were excluded from the investigation. The study was approved by the local Ethical Committees of the institutions, and all individuals signed an informed consent (CAPPesq#155/02).

Thirty four elderly bipolar patients using Li and 30 elderly bipolar patients not using Li were recruited. In addition, 25 elderly healthy subjects from the community with no psychiatric/neurological disorders were selected for the present investigation.

### Data collection

Clinical and demographic data were retrospectively collected by means of medical case note review. All the subjects and at least one close relative were interviewed by the same psychiatrist to confirm and complete the data obtained from the medical records.

The diagnosis of BD was confirmed using the Operational Criteria Checklist for Psychotic Illness (OPCRIT).^29^

The Young Mania Rating Scale (YMRS),^30^ and the Montgomery–Asberg Depression Rating Scale (MADRS)^31^ were employed to certify that bipolar subjects were euthymic (presenting scores <7 on both scales) at the time of the study.

To confirm that bipolar patients in the Li group were currently using this medication, Li blood levels were checked on the day of the MRI scanning procedure. The most recent measures of Li blood levels were also retrieved from medical records to ensure that patients had been using Li systematically.

The control group was administered a general medical questionnaire, a physical and neurological examination, and was interviewed using the Cambridge Mental Disorders of the Elderly Examination (CAMDEX)^32^ to exclude psychiatric disorders.

A longitudinal neuropsychological evaluation was also performed on all participants using the Informant Questionnaire on Cognitive Decline in the Elderly (IQCODE). The IQCODE is a rating scale of cognitive and functional decline (26 items) that compares the current performance of the subject with that of 10 years ago. Cut-off scores >3.6 are suggestive of dementia.^33, 34^

### Imaging data acquisition

MRI images were acquired with a GE Signa LX 1.5T system (Milwaukee, WI, USA). Contiguous 1.5-mm axial images across the entire brain were acquired with an axial T1-SPGR sequence providing 124 contiguous slices (voxel size 0.86 × 0.86 × 1.5 mm, echo time 5.2 ms, repetition time 12.10 ms, flip angle 20, field of view 22 cm and matrix 256 × 192).

The images were inspected by an experienced radiologist who was blinded to diagnostic status, to allow the exclusion of subjects with gross brain lesions.

### MRI image processing

For MRI image analysis, we employed voxel-based morphometry (VBM) methods^35^ using the Statistical Parametric Mapping (SPM) program (www.fil.ion.ucl.ac.uk/spm), version 2000 (SPM2). An optimized VBM protocol was applied, to take account of the structural characteristics of elderly brains. A customized MRI template was created specifically for the study, consisting on an average T1-weighted image and *a priori* GM, white matter (WM) and cerebrospinal fluid (CSF) templates, based on the images of all subjects included in the study, in order to more closely match the population under investigation and the image acquisition protocols used.^36^

To build this template, images were spatially normalized to the standard SPM T1-MRI template, based on 152 healthy subjects from the Montreal Neurological Institute (MNI).^37^ This spatial normalization step was restricted to linear 12-parameter affine transformations, to minimize deformations of our original images. Spatially normalized images were then segmented into GM, WM and CSF compartments, with a modified mixture model cluster analysis technique.^38^ This used the MNI prior probability maps provided in the SPM2 package, overlaid onto the images to classify voxels in terms of their probability of belonging to a particular tissue class. The segmentation method also included an automated brain extraction procedure to remove non-brain tissue and an algorithm to correct for image intensity non-uniformity. Finally, images were smoothed with an isotropic Gaussian kernel (8 mm full-width at half-maximum (FWHM)) and averaged to provide the GM, WM and CSF templates in stereotactic space.

The processing of the original images from all bipolar patients and controls was then carried out, beginning by image segmentation with the study-specific, *a priori* GM, WM and CSF templates. Extracted GM and WM images were then spatially normalized to the customized GM and WM templates with 12-parameter linear and non-linear (7 × 9 × 7 basis functions) transformations. The parameters resulting from this spatial normalization step were then reapplied to the original structural images. These fully normalized images were resliced with trilinear interpolation to a final voxel size of 2 × 2 × 2 mm^3^ and segmented into GM, WM and CSF partitions. Finally, images from patients and controls were smoothed with a Gaussian kernel. We prepared two set of images: one filtered using the most commonly employed size of 12 mm FWHM, and a second set filtered at 4 mm. Our decision to repeat the analysis with images filtered using a smaller, 4-mm Gaussian kernel was due to the fact that this may increase the accuracy of VBM to detect differences in small brain areas such as medial temporal structures.^39, 40^

### Statistical analysis

To investigate the presence of significant GM volumetric differences in the medial temporal region between the three groups, an overall analysis of variance (ANOVA) was initially carried out. In each analysis, a measure of the total amount of GM in the brain was entered as a confounding covariate, given by the sum of voxels within the corresponding GM compartment of each participant. Only voxels with values above an absolute threshold of 0.05 entered the analyses, resulting in a total search volume of ~250 000 voxels.

Resulting statistics were thresholded at the one-tailed *P*<0.001 level of significance (*Z*>3.09), and displayed on a statistical parametric map into standard anatomical space. The ANOVA map was searched for the presence of significant F values on the voxels contained in the hippocampi. The voxels mapped to these structures were circumscribed using the small volume correction (SVC) tool available in the SPM package, whereby we applied predefined, spatially normalized volumes of interest on the ANOVA map for the hippocampus (resulting in a search volume of 667 voxels for the left hippocampus and 677 voxels for the right hippocampus). Any GM differences within those areas were reported as significant if surviving family-wise error (FWE) correction for multiple comparisons (*P*<0.05).^41^ Only clusters with >10 voxels were considered. *Post hoc* evaluation of significant ANOVA findings in these regions was then performed with secondary two-tailed independent sample *t*-tests. Finally, we conducted voxel-wise linear correlation analyses within the Li group between hippocampal volume and Li blood levels.

In all analyses, we converted MNI coordinates of voxels of maximal statistical significance to the Talairach and Tournoux (1988) system.^42, 43^

## Results

Three bipolar patients and two elderly healthy subjects were excluded from further analyses due to movement artifacts during imaging data acquisition. An additional minority of subjects had to be excluded due to the presence of gross brain lesions detected at MRI scanning, including one patient and one control subject who presented subdural collections, one bipolar patient who had an ischemic cortical lesion in the temporal lobe and two patients presenting a frontal meningioma. The final number of MRI data sets analyzed was 57 from the bipolar sample (30 using Li and 27 not using Li) and 22 from the control group.

### Sociodemographic and clinical characteristics

The subjects' age range was 60 to 87 years (mean=68.7 years; s.d.=5.4 years) and there were no statistical differences in demographic characteristics between the groups (Table 1).

In the bipolar group, the age of onset of BD varied from 15 to 73 years (mean= 43.1, s.d.=14.5 years) and the average time of disease duration was 25.9±14.9 years. The mean number of total affective episodes during life time was 19.1±18.5, with 0.8±0.71 episodes per year on average (median=0.64). Subjects displayed a 22.6±21.9-month average time interval from the last affective episode.

No statistical differences in clinical characteristics and course of BD were observed between the groups of patients using Li and not using Li (Table 2).

However, an overall cognitive comparison between the groups using the IQCODE scores showed that the group of patients taking Li presented cognitive scores lower than the two other groups (the lower the scores, the better the cognitive performance) (Table 3).

### Use of Li

All patients in the Li group were using the medication correctly, as confirmed by the Li blood levels collected during the MRI procedure (mean=0.78±0.32 mEq l^−1^, min=0.2 mEq l^−1^, max=1.6 mEq l^−1^). The mean Li dosages used to achieve such blood levels were 761.7 mg per day (min=300 mg per day, max=1200 mg per day) during 61.7 months on average (min=12 months, max=228 months).

No significant differences in regard to the use of anti-depressants (*P*=0.80), anti-psychotics (*P*=0.89) or benzodiazepines (*P*=1.0) were observed between bipolar patients using and not using Li (Table 4).

Of the 27 patients not taking Li at the moment of the study, 18 patients (66.7%) had been treated with this drug in the past. They had not been using Li for 45 months on average (min=8 months, max=180 months).

### Neuroimaging findings

The sum of GM voxels in the segmented images totaled 61 502.99±5434.55 in the Li group, 58 607.32±4841.89 in the non-Li group and 62 321.29±6440.11 in healthy control subjects (F=3.17, *P*=0.047). *Post hoc* independent *t*-tests indicated significantly decreased global GM in the non-Li group compared with the other two groups (Li versus non-Li: *t*=2.11, *P*=0.039; control group versus non-Li: *t*=2304, *P*=0.026; control group versus Li; *t*=0.49, *P*=0.63).

**Figure 1:**
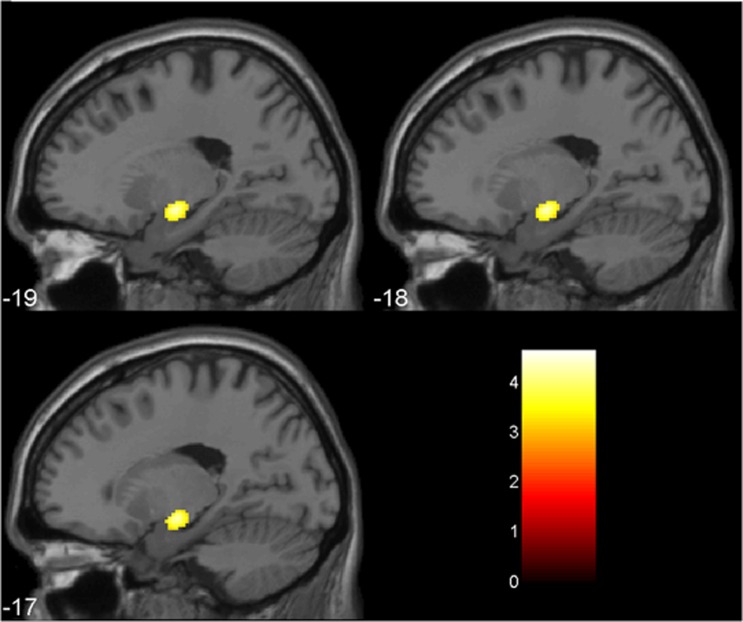
A focus of increased gray matter volume in bipolar disorder subjects using lithium (Li) (*n*=27) compared with non-Li-treated (non-Li) patients (*n*=30) is shown in the left hippocampus, highlighted in yellow (at the *Z*>3.09 cutoff, uncorrected for multiple comparisons and using an extent threshold of 10 voxels). Findings are overlaid on sagittal brain slices spatially normalized into an approximation to the Talairach and Tournoux stereotactic atlas (Talairach and Tornoux, 1988), and the numbers associated with each frame represent standard coordinates in the *x*-axis. This cluster of between-group gray matter difference retained statistical significance at the *P*<0.05 level after family-wise error correction for multiple comparisons using the small volume correction (SVC) tool in Statistical Parametric Mapping (SPM). Statistical details are provided in Table 3, including the coordinates of the voxel of maximal statistical significance and its peak *Z*-score.

In the VBM analysis of the medial temporal region using images filtered with a 12-mm Gaussian kernel, the ANOVA map comparing the three groups (entering total GM in the brain as a confounder) indicated the presence of one voxel cluster of statistical significance located in the left hippocampus (31 voxels, SVC-based pFWE=0.010, peak coordinates_*x*,*y*,*z*_= (−16 −10 −10), F=10.44, *Z*=3.72). *Post hoc* unpaired *t*-tests showed a reduction of volume in the left hippocampus in the non-Li group relative both to the Li group (51 voxels, SVC-based pFWE=0.002, peak coordinates_*x*,*y*,*z*_=(−18 −10 −10), *T*=4.34, *Z*=4.09) and to the control group (24 voxels, SVC-based pFWE=0.010, peak coordinates_*x*,*y*,*z*_= (−16 −12 −11), *T*=3.83, *Z*=3.65). An additional exploratory search for volumetric differences in the right medial temporal region using unpaired *t*-tests indicated a reduction of volume in the right hippocampus in the non-Li group relative to the control group (16 voxels, SVC-based pFWE=0.035, peak coordinates_*x*,*y*,*z*_= (34 −37 −2), *T*=3.39, *Z*=3.26), as well as a weak trend toward reduced volume in the right hippocampus in the non-Li group versus the Li group (*P*<0.01 uncorrected, peak coordinates_*x*,*y*,*z*_=(14 −31 11), *T*=2.66, *Z*=2.59). There were no significant volume differences in the left or right hippocampus between the Li-treated group and controls. In the same VBM comparisons of the medial temporal region using images filtered with the 4-mm Gaussian kernel, we found the same foci of statistical difference between groups in the hippocampus (Table 5 and Figure 1).

**Figure 2:**
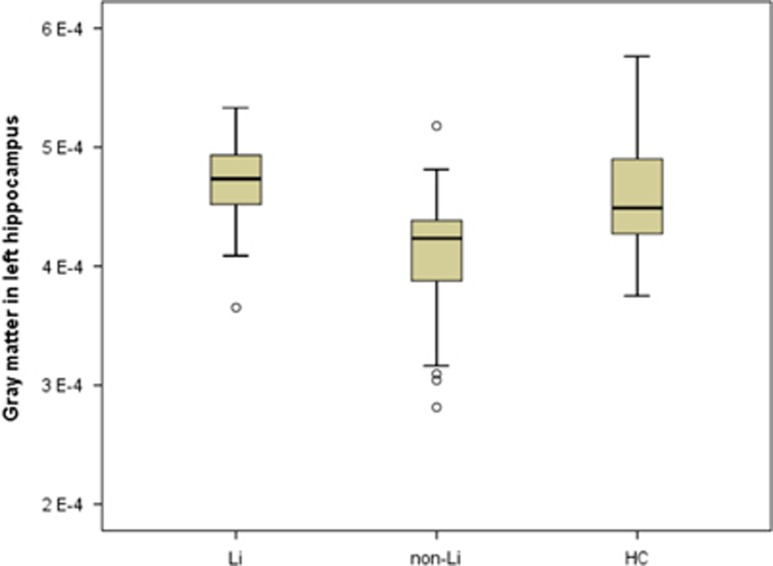
Scatter plot of the left hippocampal gray matter volume (normalized to the total sum of gray matter voxels in the brain) in the lithium (Li), non-lithium (non-Li) and healthy control (HC) groups. Hippocampal measures were taken from the voxel of peak statistical difference between the three groups (peak coordinates_*x*,*y*,*z*_=−16 −10 −10, F=10.44, *Z*=3.72). Left hippocampal volumes in the non-Li-treated group were significantly lower compared with both the Li and HC groups (see statistical details in Table 4).

Figure 2 shows a box plot of the left hippocampus (GM volume for the voxel of peak statistical difference normalized to the total amount of GM in the brain) for all subjects in the three groups (data Gaussian-filtered with 12 mm).

When we repeated the analysis using an analysis of covariance model controlling for the possible confounding effects of age, gender and number of affective episodes, the focus of between-group statistical difference in the left hippocampus retained a statistically significant trend (*P*<0.066, with SVC-based pFWE correction for multiple comparisons; data Gaussian-filtered with 12 mm). *Post hoc* unpaired *t*-tests showed similar findings as reported above, with a reduction of volume in the left hippocampus in the non-Li group relative to both the Li group (29 voxels, SVC-based pFWE=0.010, peak coordinates_*x*,*y*,*z*_=(−18 −8 −12), *T*=3.87, *Z*=3.67) and the control group (4 voxels, SVC-based pFWE=0.039, peak coordinates_*x*,*y*,*z*_=(−16−10−10), *T*=3.35, *Z*=3.22).

We also we repeated the above VBM analysis including not only gender but also years of education as an additional confounding covariate. The focus of between-group statistical difference in the left hippocampus remained statistically significant (*P*<0.018, with SVC-based pFWE correction for multiple comparisons; data Gaussian-filtered with 12 mm). *Post hoc* unpaired *t*-tests showed again reduced volume of the left hippocampus in the non-Li group relative to both the Li group (42 voxels, SVC-based pFWE=0.003, peak coordinates_*x*,*y*,*z*_=(−18 −10 −10), *T*=4.21, *Z*=3.97) and the control group (10 voxels, SVC-based pFWE=0.021, peak coordinates_*x*,*y*,*z*_=(−16 −12 −11), *T*=3.57, *Z*=3.42).

Finally, the linear correlation analysis within the Li group between hippocampal volumes and Li blood levels showed no significant findings.

## Discussion

To the best of our knowledge, this is the first neuroimaging investigation showing that elderly bipolar patients using Li carbonate for >1 year presented larger volume in their left hippocampus when compared with BD patients not taking Li for at least 8 months. The demographic and clinical characteristics of the BD patients did not show statistically significant differences between the groups, but the IQCODE scores showed that the group of patients taking Li presented cognitive scores lower than the other two groups (better cognition performance), reinforcing the fact that the neuroimaging findings are probably due to Li itself and not secondary to clinical differences. Even the characteristics that were different between the groups, but not statistically different, such as gender and number of affective episodes, did not exert effects on the neuroimaging findings as shown by analysis of covariance.

The hippocampus is thought to be among the most important brain structures that display volumetric alterations in BD,^44, 45^ although brain imaging findings pertaining to the hippocampal structure in BD are somehow controversial. Pantelis *et al.*^46^ found reductions of GM volume in the left hippocampus after onset of psychosis,^46^ and Moorhead *et al.*^47^ observed progressive loss of GM volume in the left hippocampus and fusiform gyrus in BD patients.^47^ Yucelet *et al.*^48^ found progressive increased volume of the hippocampus after treatment with Li, in association with improved verbal memory.^20^ These findings suggest that treatment with Li at least may reverse losses of hippocampal GM volume, which is consistent with our data.

Recent studies, with younger populations have also reported neuroprotective effects in brain morphology of Li treatment. Germaná *et al.*,^49^ in a meta-analysis, reported that GM in the subgenual anterior cingulated gyrus on the right (extending in to the hypothalamus) and in the postcentral gyrus, the hippocampus/amygdala complex and the insula on the left was greater in BD patients on Li treatment compared with all other treatment groups. Hajek *et al.*^50^ performed a meta-analysis of neuroimaging studies that subdivided patients based on the presence or absence of current Li treatment from a database of 16 studies (346 BD patients). Both the left- and right-hippocampal volumes were significantly larger in the Li group than in controls or the non-Li group, which had smaller left- and right-hippocampal volumes than the control group.^50^

We can hypothesize that the longer-term use of Li by elderly bipolar patients in our sample could have a neuroprotective effect in the hippocampal region against continuous injuries due to repetitive affective episodes, leading to an increased volume in the left hippocampus in the group of patients using this drug. In fact, *in vivo* studies have shown that Li exerts neuroprotective effects against a variety of cell injuries.^51, 52^ Neurogenesis does occur in restricted human brain regions, especially in the subventricular zone and the subgranular layer of the hippocampus.^53^ Chen *et al.*^23^ observed a significant increase of dividing cells in the hippocampus of rodents that were treated with Li for 4 weeks.^14^ This effect was also noted by Son *et al.*,^54^ with an increase of ~54% in hippocampal cells of rodents treated with Li compared with those not treated, but acute treatment (2 days) did not reveal this neurogenesis effect.^54^ More recently, Zanni *et al.*^55^ reported that an *in vitro* model of hippocampal neurogenesis in the juvenile brain showed that neural stem/progenitor cells are protected by Li, as reflected by higher proliferation rates without a reduction in apoptosis.^55^ However, Lueke *et al.*^56^ in an *in vitro* model of presynaptic function of primary hippocampal neurons (the release and reuptake of pool vesicles) did not observe statistically significant changes using normal and high Li concentrations under acute and chronic Li treatment/exposures, indicating that the previous observation of changes in hippocampal volume may be due to postsynaptic effect of Li. Indeed, there are studies suggesting the existence of independent mechanisms related to presynaptic and postsynaptic activities.^57, 58^

The molecular mechanism underlying the effects of Li in BD still remains unknown, despite several studies shedding light on some of Li's potential mechanisms, such as its action in GSK3-signaling, in the inhibition of inositol monophosphate and its role in extracellular-regulated kinase and in GABAergic transmission via gephyrin phosphorylation.^58, 59, 60^ However, it is still unclear whether Li-mediated adaptations can be exclusively attributed to these mechanisms.

Using the IQCODE measurements, it was interesting to observe that the elderly bipolar patients taking Li presented better cognitive functioning not only when compared with the non-Li group, but also with the control group. It should also be noted that none of the BD patients included in our study had cognitive deficits within the range compatible with a diagnosis of dementia. Therefore, it is unlikely that our comparison of hippocampal volumes would have been biased by the inclusion of individuals with disproportionate cognitive deficits (presumably associated with reduced hippocampal volumes unrelated to the use of mood stabilizers or the diagnosis of BD).

VBM is an automated technique that detects regionally specific differences in brain tissue composition on a voxel-by-voxel basis. Its use has become widespread, being less labor intensive and less susceptible to examiner bias as compared with conventional manual region-of-interest-based morphometric methods. Despite these advantages, VBM has some limitations. Imperfections in spatial normalization could have implications for the sensibility of the technique to detect changes in regions of high anatomical variance. To avoid this bias, we used a customized template based on our own MRI images, which matched more closely the elderly population under study, and led to a lesser degree of image deformation during the process of spatial normalization than would have occurred had we used the standard SPM template (which is based on young healthy subjects not displaying ventricle dilatation). Such a strategy is likely to have improved the spatial specificity of our analysis, minimizing the chance of significant findings being confounded by poor image normalization.^36^ A further possible limitation of VBM is the difficulty in detecting volumetric differences in small brain regions such as medial temporal structures. We used a smaller smoothing kernel (4-mm isotropic FWHM Gaussian kernel rather than the 12 mm) and the SVC tool to evaluate possible differences in the hippocampus. Such methodology increases the accuracy of VBM in detecting differences, even in small areas such as the hippocampal region.^39, 40, 54^ Neuroimaging studies comparing VBM methods (using small smoothing kernel and SVC) with conventional manual region-of-interest analyses showed that both methods are equally sensitive to identifying possible differences in the volume of the hippocampus.^39, 40, 61^

Others techniques and methods in neuroimaging are being used to identify differences between the bipolar patient under treatment with Li and bipolar patients using other medications but not Li. Using the diffusion tensor imaging technique, MRI investigations have documented changes in WM microstructure in BD samples using mood stabilizers.^62^ In one of the most recent of such diffusion tensor imaging studies, bipolar subjects with longer duration of Li treatment displayed higher WM integrity.^63^ Future multimodal MRI studies are warranted to evaluate how such WM features may be related to the GM volume changes associated with Li usage, as reported herein.

The present study has both strengths and limitations. Particular strengths include the novelty of a neuroimaging investigation of elderly bipolar patients evaluating the effects of longer-term Li treatment over the volumes of specific areas in the brain related to memory function. However, replication studies using a larger sample size and the inclusion of other clinical assessments (for example, IQ testing) would confirm and refine the preliminary results observed in this study.

After the first rediscovery of Li by John Cade more than 50 years ago, the recent increased evidence of the possible neuroprotective action of this drug could be responsible for the second rediscovery of Li. If our findings are confirmed by further studies, including ones using prospective sample designs, psychiatrists could prescribe Li for a patient with BD not only for its effect as a mood stabilizer, but also as a prophylactic medication against cognitive decline and dementia in predisposed patients. New perspectives on its clinical use in the treatment of acute neuronal injuries and chronic neurodegenerative diseases should also be considered.

## Figures and Tables

**Table 1:** Demographic characteristics of elderly subjects (*n*=79)

	*Elderly bipolar patients*			
	*Lithium*	*Non-lithium*	*Elderly controls*	P*-value*
*N*	30	27	22	
Sex (%)(M/F)	43.3/56.7	22.2/77.8	26/74	0.19^a^
Age (years)—mean (s.d.)	67.9 (4.6)	70.2 (4.9)	67.9 (6.7)	0.23^b^
				
*Marital status (%)*				0.27^c^
Married	60	29.6	56.5	
Widowed	16.7	40.7	17.4	
Divorced	13.3	18.5	13	
Single	10	11	13	
Years of study—mean (s.d.)	6.6 (5.1)	6.3 (4.5)	9.2 (5.6)	0.10^b^
				
*Ethnicity*				0.35^c^
European descendant	83.3	85.2	82.6	
Mixed (European and African descendants)	13.3	7.4	0	
African descendant	0	3.7	4.3	
East Asian descendant	3.3	7.4	13	

Abbreviations: ANOVA, analysis of variance; F, female; M, male.

a *X*^2^-test.

b ANOVA.

c Fisher test.

**Table 2:** Clinical characteristics of bipolar patients using and not using lithium

	*Lithium (*n*=30)*	*Non-lithium (*n*=27)*	P*-value*
Age of onset (years)—mean (s.d.)	44.6 (13.1)	41.4 (15.9)	0.4^a^
Number of affective episodes—mean (s.d.)	14.7 (16.1)	24.0 (20.1)	0.07^a^
Episodes per year— mean (s.d.)	0.73 (0.68)	0.89 (0.74)	0.27^a^
Depressive episodes—mean (s.d.)	7.9 (8.6)	14.0 (14.0)	0.17^a^
Maniac episodes—mean (s.d.)	6.9 (8.7)	10.2 (10.4)	0.13^b^
Duration of disease (years)—mean (s.d.)	23.4 (12.7)	28.9 (16.8)	0.16^a^
Psychotic features (%)	53.3	66.7	0.31^b^
Suicide attempt (%)	23.3	22.2	0.93^b^
Remission (months)—mean (s.d.)	24.7 (23.8)	20.2 (19.7)	0.45^a^

a Student's *t*-test.

b *X*^2^-test.

**Table 3:** Psychiatric medication used by the patients using and not using lithium (*n*=57)

	*Lithium (*n*=30)*	*Non-lithium (*n*=27)*
*Antipsychotic drugs (*n*/%)*	6/20	5/18.5
Olanzapine	2	1
Risperidone	4	4
		
*Benzodiazepines (*n*/%)*	10/33.3	9/33.3
Clonazepam depressive episodes (mean)	8	7
Diazepam	0	1
Flunitrazepam	1	1
Lorazepam	1	0
		
*Antidepressants (*n*/%)*	6/20	5/18.5
Sertraline	3	0
Fluoxetine	0	1
Citalopram	0	1
Tranylcypromine	1	1
Imipramine	1	0
Nortriptyline	1	1
Mirtazapine	1^a^	1

a One patient was taking a combination of Sertaline and Mirtazapine.

**Table 4:** IQCODE scores of all participants (*n*=79)

	*Number of participants*	*Mean*	*s.d.*	*Median*	P*-value*
Bipolar lithium	30	3.04	0.43	3.05	0.04^a^
Bipolar non-lithium	27	3.31	0.49	3.31	
Controls	22	3.12	0.21	3.07	
Total	79	3.16	0.42	3.10	

Abbreviation: ANOVA, analysis of variance.

a ANOVA.

**Table 5:** Significant hippocampal volume differences between elderly bipolar patients using Li, elderly bipolar patients not using Li (non-Li) and elderly HC

*Comparison*	*Region*	*F-value*^a^	T*-value*^b^	Z*-value*^c^	P*-value*^d^	*Size of cluster*^e^	*Coordinates (*x*,*y*,*z*)*^f^
ANOVA	Left hippocampus^g^	10.44		3.72	0.010	31	−16 −10 −10
							
Post hoc t*-tests*							
Reduction in non-Li group relative to Li group	Left hippocampus^g^		4.34	4.09	0.002	51	−18 −10 −10
Reduction in non-Li group relative to control group	Left hippocampus^g^		3.83	3.65	0.010	24	−16 −12 −11

Abbreviations: ANOVA, analysis of variance; HC, healthy controls; Li, lithium; SVC, small volume correction.

a F-value (ANOVA comparison between the three groups) for the voxel of maximal statistical significance within each cluster.

b *T*-value (*Post hoc* tests) for the voxel of maximal statistical significance within each cluster.

c *Z*-scores for the voxel of maximal statistical significance within each cluster.

d Statistical significance after correction for multiple comparisons; inferences were made at the level of individual voxels (family-wise error correction) and a minimum extent threshold of 10 voxels.

e Number of contiguous voxels that surpassed the initial threshold of *P*<0.001 (uncorrected) in the statistical parametric maps.

f Talairach and Tornoux (1988) coordinates of the voxel of maximal statistical significance within each cluster.

g Brain region of statistical significance using SVC tool.
